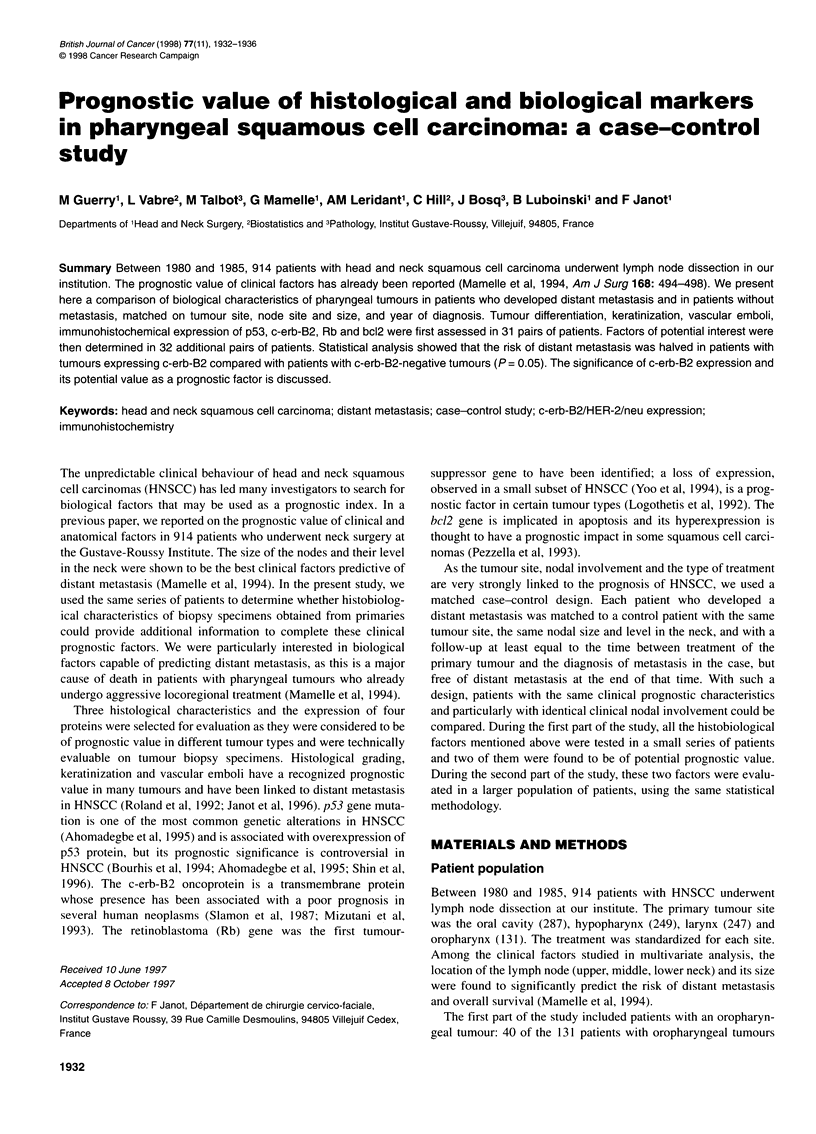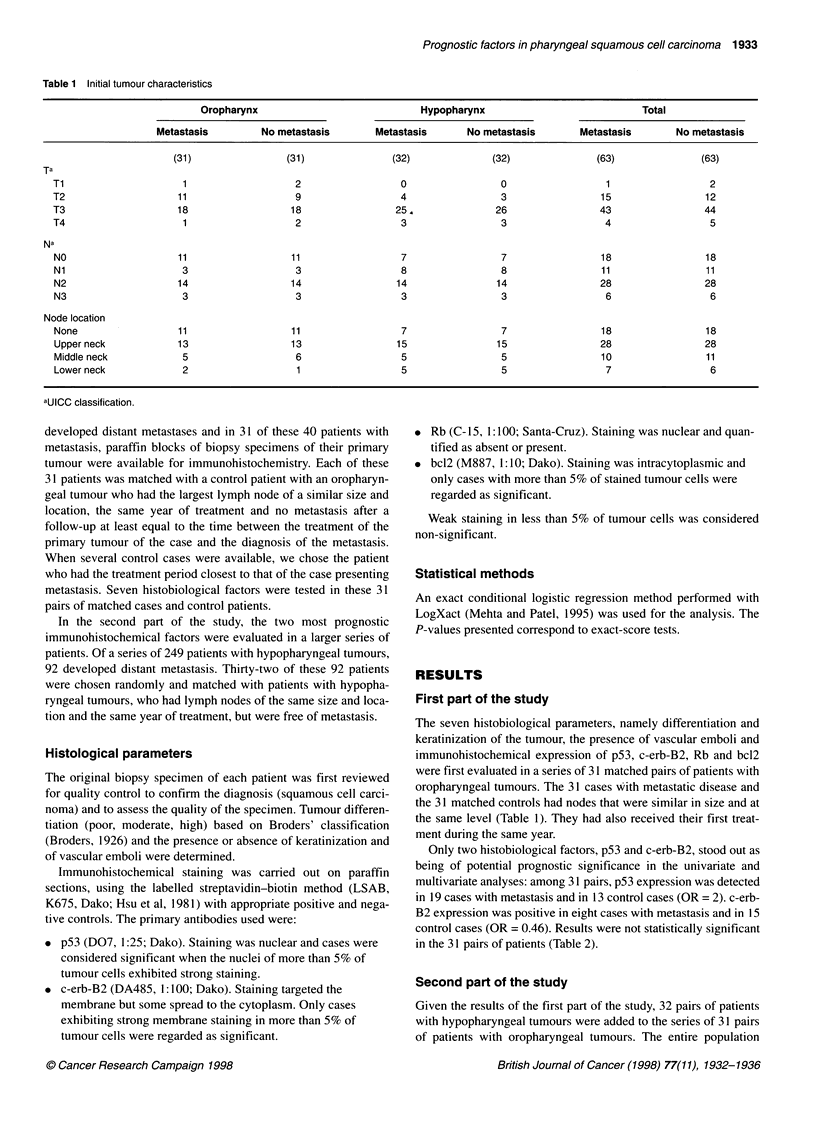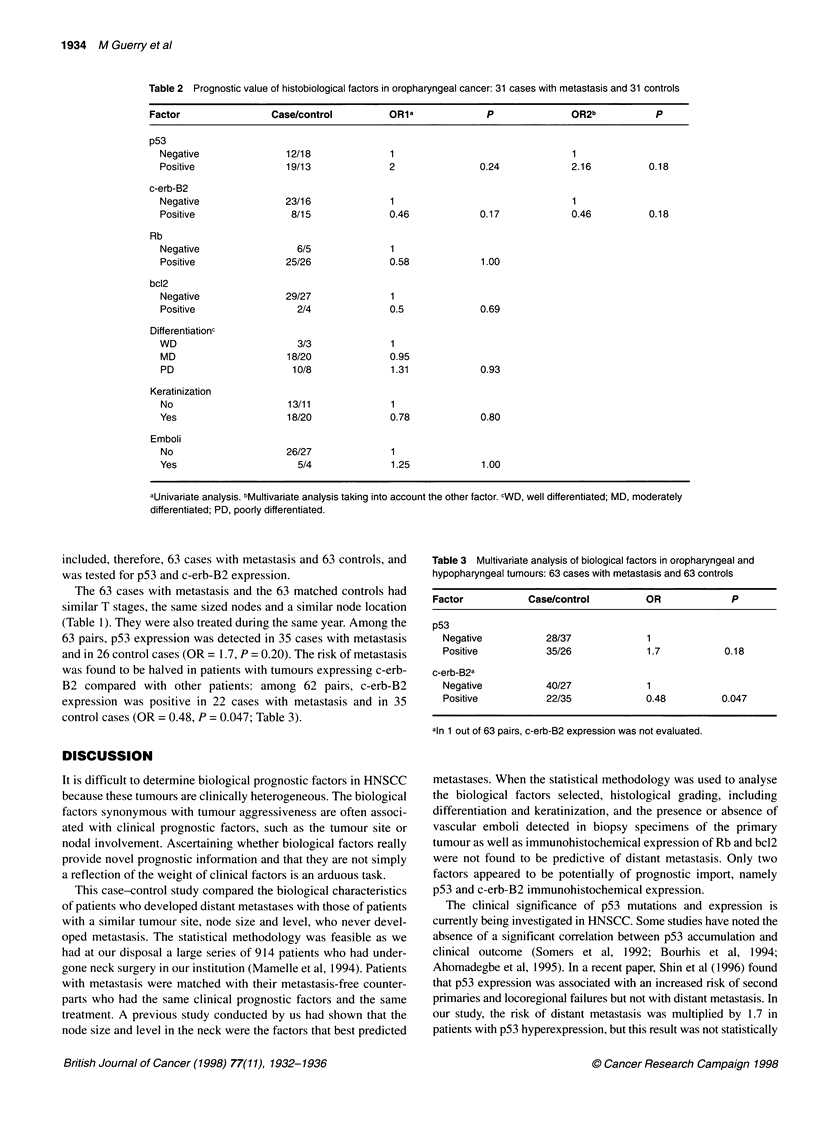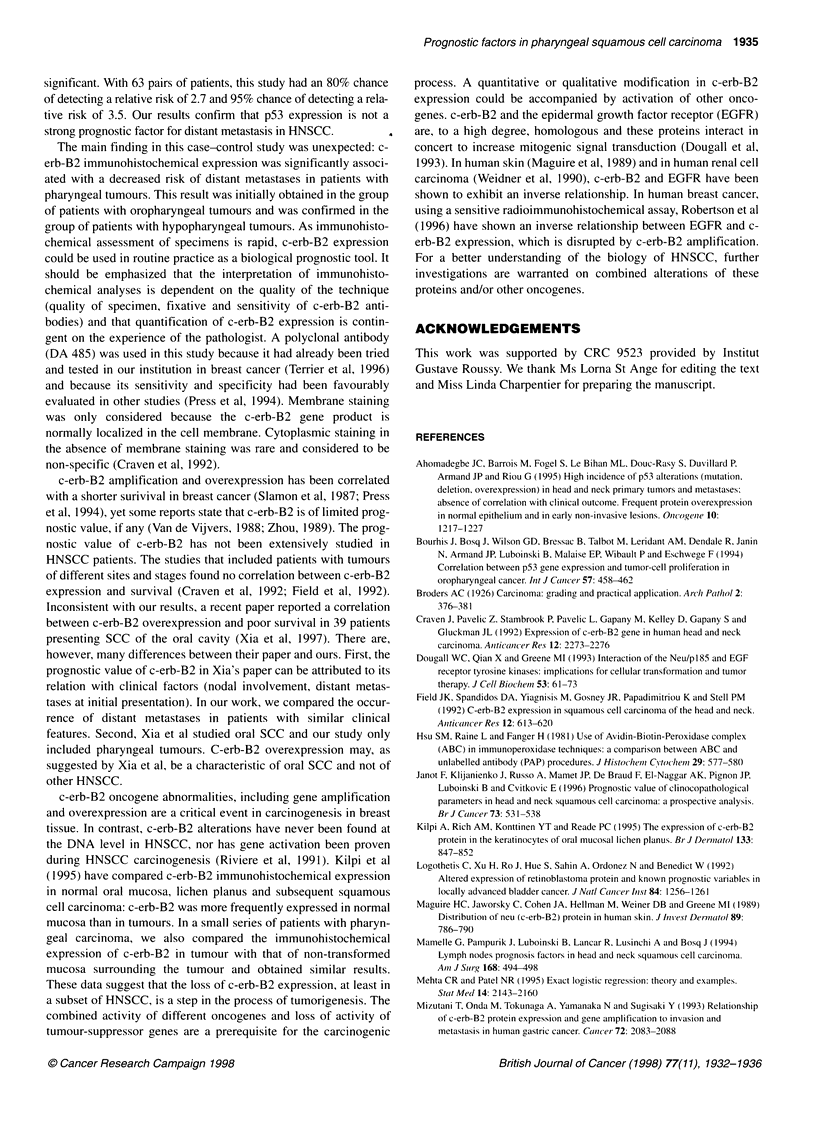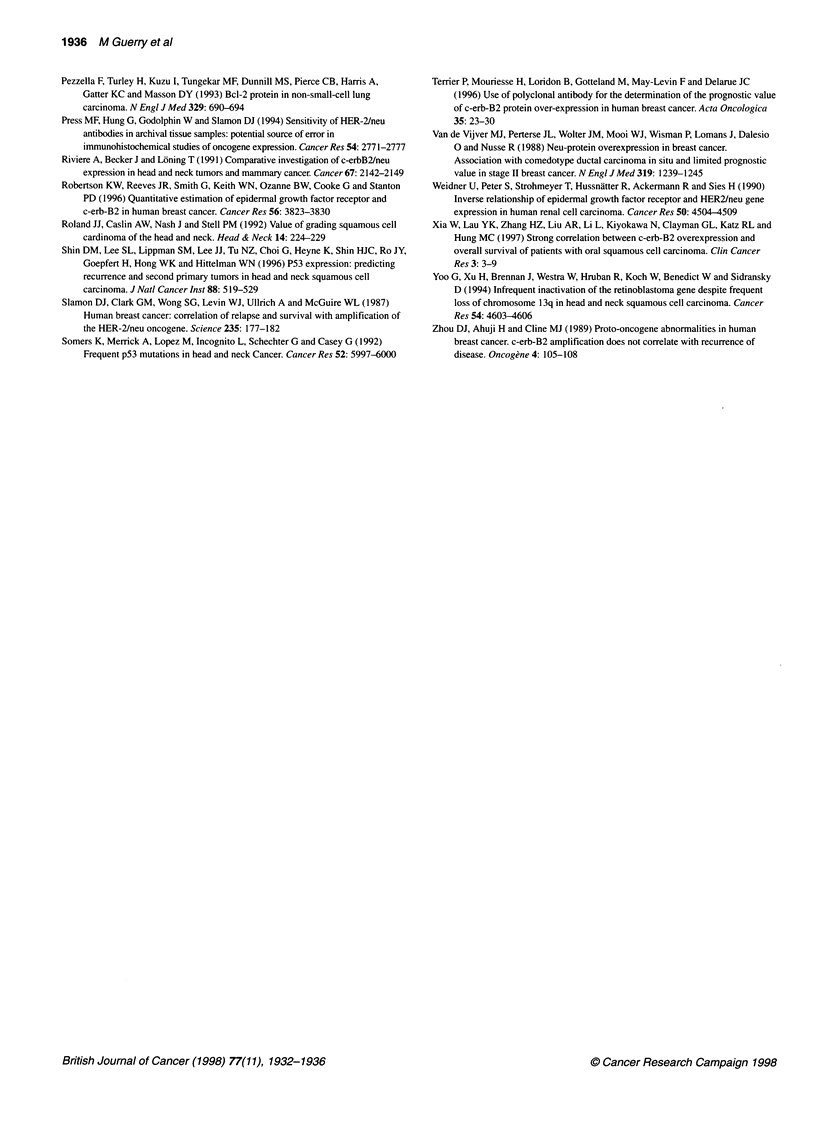# Prognostic value of histological and biological markers in pharyngeal squamous cell carcinoma: a case-control study.

**DOI:** 10.1038/bjc.1998.320

**Published:** 1998-06

**Authors:** M. Guerry, L. Vabre, M. Talbot, G. Mamelle, A. M. Leridant, C. Hill, J. Bosq, B. Luboinski, F. Janot

**Affiliations:** Department of Head and Neck Surgery, Institut Gustave-Roussy, Villejuif, France.

## Abstract

Between 1980 and 1985, 914 patients with head and neck squamous cell carcinoma underwent lymph node dissection in our institution. The prognostic value of clinical factors has already been reported (Mamelle et al, 1994, Am J Surg 168: 494-498). We present here a comparison of biological characteristics of pharyngeal tumours in patients who developed distant metastasis and in patients without metastasis, matched on tumour site, node site and size, and year of diagnosis. Tumour differentiation, keratinization, vascular emboli, immunohistochemical expression of p53, c-erb-B2, Rb and bcl2 were first assessed in 31 pairs of patients. Factors of potential interest were then determined in 32 additional pairs of patients. Statistical analysis showed that the risk of distant metastasis was halved in patients with tumours expressing c-erb-B2 compared with patients with c-erb-B2-negative tumours (P = 0.05). The significance of c-erb-B2 expression and its potential value as a prognostic factor is discussed.


British Journal of Cancer (1998) 77(11), 1932–1936
© 1998 Cancer Research Campaign

### Prognostic value of histological and biological markers in pharyngeal squamous cell carcinoma: a case–control study

**M Guerry[1], L Vabre[2], M Talbot[3], G Mamelle[1], AM Leridant[1], C Hill[2], J Bosq[3], B Luboinski[1] and F Janot[1]**

Departments of [1]Head and Neck Surgery, [2]Biostatistics and [3]Pathology, Institut Gustave-Roussy, Villejuif, 94805, France

**Summary** Between 1980 and 1985, 914 patients with head and neck squamous cell carcinoma underwent lymph node dissection in our institution. The prognostic value of clinical factors has already been reported (Mamelle et al, 1994, *Am J Surg* **168:** 494–498). We present here a comparison of biological characteristics of pharyngeal tumours in patients who developed distant metastasis and in patients without metastasis, matched on tumour site, node site and size, and year of diagnosis. Tumour differentiation, keratinization, vascular emboli, immunohistochemical expression of p53, c-erb-B2, Rb and bcl2 were first assessed in 31 pairs of patients. Factors of potential interest were then determined in 32 additional pairs of patients. Statistical analysis showed that the risk of distant metastasis was halved in patients with tumours expressing c-erb-B2 compared with patients with c-erb-B2-negative tumours ($P = 0.05$). The significance of c-erb-B2 expression and its potential value as a prognostic factor is discussed.

**Keywords:** head and neck squamous cell carcinoma; distant metastasis; case–control study; c-erb-B2/HER-2/neu expression; immunohistochemistry

The unpredictable clinical behaviour of head and neck squamous cell carcinomas (HNSCC) has led many investigators to search for biological factors that may be used as a prognostic index. In a previous paper, we reported on the prognostic value of clinical and anatomical factors in 914 patients who underwent neck surgery at the Gustave-Roussy Institute. The size of the nodes and their level in the neck were shown to be the best clinical factors predictive of distant metastasis (Mamelle et al, 1994). In the present study, we used the same series of patients to determine whether histobiological characteristics of biopsy specimens obtained from primaries could provide additional information to complete these clinical prognostic factors. We were particularly interested in biological factors capable of predicting distant metastasis, as this is a major cause of death in patients with pharyngeal tumours who already undergo aggressive locoregional treatment (Mamelle et al, 1994).

Three histological characteristics and the expression of four proteins were selected for evaluation as they were considered to be of prognostic value in different tumour types and were technically evaluable on tumour biopsy specimens. Histological grading, keratinization and vascular emboli have a recognized prognostic value in many tumours and have been linked to distant metastasis in HNSCC (Roland et al, 1992; Janot et al, 1996). *p53* gene mutation is one of the most common genetic alterations in HNSCC (Ahomadegbe et al, 1995) and is associated with overexpression of p53 protein, but its prognostic significance is controversial in HNSCC (Bourhis et al, 1994; Ahomadegbe et al, 1995; Shin et al, 1996). The c-erb-B2 oncoprotein is a transmembrane protein whose presence has been associated with a poor prognosis in several human neoplasms (Slamon et al, 1987; Mizutani et al, 1993). The retinoblastoma (Rb) gene was the first tumour-suppressor gene to have been identified; a loss of expression, observed in a small subset of HNSCC (Yoo et al, 1994), is a prognostic factor in certain tumour types (Logothetis et al, 1992). The *bcl2* gene is implicated in apoptosis and its hyperexpression is thought to have a prognostic impact in some squamous cell carcinomas (Pezzella et al, 1993).

As the tumour site, nodal involvement and the type of treatment are very strongly linked to the prognosis of HNSCC, we used a matched case–control design. Each patient who developed a distant metastasis was matched to a control patient with the same tumour site, the same nodal size and level in the neck, and with a follow-up at least equal to the time between treatment of the primary tumour and the diagnosis of metastasis in the case, but free of distant metastasis at the end of that time. With such a design, patients with the same clinical prognostic characteristics and particularly with identical clinical nodal involvement could be compared. During the first part of the study, all the histobiological factors mentioned above were tested in a small series of patients and two of them were found to be of potential prognostic value. During the second part of the study, these two factors were evaluated in a larger population of patients, using the same statistical methodology.

*Received 10 June 1997*
*Accepted 8 October 1997*

*Correspondence to:* F Janot, Département de chirurgie cervico-faciale, Institut Gustave Roussy, 39 Rue Camille Desmoulins, 94805 Villejuif Cedex, France

### MATERIALS AND METHODS

### Patient population

Between 1980 and 1985, 914 patients with HNSCC underwent lymph node dissection at our institute. The primary tumour site was the oral cavity (287), hypopharynx (249), larynx (247) and oropharynx (131). The treatment was standardized for each site. Among the clinical factors studied in multivariate analysis, the location of the lymph node (upper, middle, lower neck) and its size were found to significantly predict the risk of distant metastasis and overall survival (Mamelle et al, 1994).

The first part of the study included patients with an oropharyngeal tumour: 40 of the 131 patients with oropharyngeal tumours

**Table 1** Initial tumour characteristics

| | Oropharynx | | Hypopharynx | | Total | |
|---|---|---|---|---|---|---|
| | Metastasis | No metastasis | Metastasis | No metastasis | Metastasis | No metastasis |
| | (31) | (31) | (32) | (32) | (63) | (63) |
| T[a] | | | | | | |
| T1 | 1 | 2 | 0 | 0 | 1 | 2 |
| T2 | 11 | 9 | 4 | 3 | 15 | 12 |
| T3 | 18 | 18 | 25 | 26 | 43 | 44 |
| T4 | 1 | 2 | 3 | 3 | 4 | 5 |
| N[a] | | | | | | |
| N0 | 11 | 11 | 7 | 7 | 18 | 18 |
| N1 | 3 | 3 | 8 | 8 | 11 | 11 |
| N2 | 14 | 14 | 14 | 14 | 28 | 28 |
| N3 | 3 | 3 | 3 | 3 | 6 | 6 |
| Node location | | | | | | |
| None | 11 | 11 | 7 | 7 | 18 | 18 |
| Upper neck | 13 | 13 | 15 | 15 | 28 | 28 |
| Middle neck | 5 | 6 | 5 | 5 | 10 | 11 |
| Lower neck | 2 | 1 | 5 | 5 | 7 | 6 |

[a]UICC classification.

developed distant metastases and in 31 of these 40 patients with metastasis, paraffin blocks of biopsy specimens of their primary tumour were available for immunohistochemistry. Each of these 31 patients was matched with a control patient with an oropharyngeal tumour who had the largest lymph node of a similar size and location, the same year of treatment and no metastasis after a follow-up at least equal to the time between the treatment of the primary tumour of the case and the diagnosis of the metastasis. When several control cases were available, we chose the patient who had the treatment period closest to that of the case presenting metastasis. Seven histobiological factors were tested in these 31 pairs of matched cases and control patients.

In the second part of the study, the two most prognostic immunohistochemical factors were evaluated in a larger series of patients. Of a series of 249 patients with hypopharyngeal tumours, 92 developed distant metastasis. Thirty-two of these 92 patients were chosen randomly and matched with patients with hypopharyngeal tumours, who had lymph nodes of the same size and location and the same year of treatment, but were free of metastasis.

### Histological parameters

The original biopsy specimen of each patient was first reviewed for quality control to confirm the diagnosis (squamous cell carcinoma) and to assess the quality of the specimen. Tumour differentiation (poor, moderate, high) based on Broders' classification (Broders, 1926) and the presence or absence of keratinization and of vascular emboli were determined.

Immunohistochemical staining was carried out on paraffin sections, using the labelled streptavidin–biotin method (LSAB, K675, Dako; Hsu et al, 1981) with appropriate positive and negative controls. The primary antibodies used were:

- p53 (DO7, 1:25; Dako). Staining was nuclear and cases were considered significant when the nuclei of more than 5% of tumour cells exhibited strong staining.
- c-erb-B2 (DA485, 1:100; Dako). Staining targeted the membrane but some spread to the cytoplasm. Only cases exhibiting strong membrane staining in more than 5% of tumour cells were regarded as significant.
- Rb (C-15, 1:100; Santa-Cruz). Staining was nuclear and quantified as absent or present.
- bcl2 (M887, 1:10; Dako). Staining was intracytoplasmic and only cases with more than 5% of stained tumour cells were regarded as significant.

Weak staining in less than 5% of tumour cells was considered non-significant.

### Statistical methods

An exact conditional logistic regression method performed with LogXact (Mehta and Patel, 1995) was used for the analysis. The *P*-values presented correspond to exact-score tests.

### RESULTS

### First part of the study

The seven histobiological parameters, namely differentiation and keratinization of the tumour, the presence of vascular emboli and immunohistochemical expression of p53, c-erb-B2, Rb and bcl2 were first evaluated in a series of 31 matched pairs of patients with oropharyngeal tumours. The 31 cases with metastatic disease and the 31 matched controls had nodes that were similar in size and at the same level (Table 1). They had also received their first treatment during the same year.

Only two histobiological factors, p53 and c-erb-B2, stood out as being of potential prognostic significance in the univariate and multivariate analyses: among 31 pairs, p53 expression was detected in 19 cases with metastasis and in 13 control cases (OR = 2). c-erb-B2 expression was positive in eight cases with metastasis and in 15 control cases (OR = 0.46). Results were not statistically significant in the 31 pairs of patients (Table 2).

### Second part of the study

Given the results of the first part of the study, 32 pairs of patients with hypopharyngeal tumours were added to the series of 31 pairs of patients with oropharyngeal tumours. The entire population

© Cancer Research Campaign 1998

**Table 2** Prognostic value of histobiological factors in oropharyngeal cancer: 31 cases with metastasis and 31 controls

| Factor | Case/control | OR1[a] | *P* | OR2[b] | *P* |
|---|---|---|---|---|---|
| p53 | | | | | |
| Negative | 12/18 | 1 | | 1 | |
| Positive | 19/13 | 2 | 0.24 | 2.16 | 0.18 |
| c-erb-B2 | | | | | |
| Negative | 23/16 | 1 | | 1 | |
| Positive | 8/15 | 0.46 | 0.17 | 0.46 | 0.18 |
| Rb | | | | | |
| Negative | 6/5 | 1 | | | |
| Positive | 25/26 | 0.58 | 1.00 | | |
| bcl2 | | | | | |
| Negative | 29/27 | 1 | | | |
| Positive | 2/4 | 0.5 | 0.69 | | |
| Differentiation[c] | | | | | |
| WD | 3/3 | 1 | | | |
| MD | 18/20 | 0.95 | | | |
| PD | 10/8 | 1.31 | 0.93 | | |
| Keratinization | | | | | |
| No | 13/11 | 1 | | | |
| Yes | 18/20 | 0.78 | 0.80 | | |
| Emboli | | | | | |
| No | 26/27 | 1 | | | |
| Yes | 5/4 | 1.25 | 1.00 | | |

[a]Univariate analysis. [b]Multivariate analysis taking into account the other factor. [c]WD, well differentiated; MD, moderately differentiated; PD, poorly differentiated.

included, therefore, 63 cases with metastasis and 63 controls, and was tested for p53 and c-erb-B2 expression.

The 63 cases with metastasis and the 63 matched controls had similar T stages, the same sized nodes and a similar node location (Table 1). They were also treated during the same year. Among the 63 pairs, p53 expression was detected in 35 cases with metastasis and in 26 control cases (OR = 1.7, $P = 0.20$). The risk of metastasis was found to be halved in patients with tumours expressing c-erb-B2 compared with other patients: among 62 pairs, c-erb-B2 expression was positive in 22 cases with metastasis and in 35 control cases (OR = 0.48, $P = 0.047$; Table 3).

**Table 3** Multivariate analysis of biological factors in oropharyngeal and hypopharyngeal tumours: 63 cases with metastasis and 63 controls

| Factor | Case/control | OR | *P* |
|---|---|---|---|
| p53 | | | |
| Negative | 28/37 | 1 | |
| Positive | 35/26 | 1.7 | 0.18 |
| c-erb-B2[a] | | | |
| Negative | 40/27 | 1 | |
| Positive | 22/35 | 0.48 | 0.047 |

[a]In 1 out of 63 pairs, c-erb-B2 expression was not evaluated.

### DISCUSSION

It is difficult to determine biological prognostic factors in HNSCC because these tumours are clinically heterogeneous. The biological factors synonymous with tumour aggressiveness are often associated with clinical prognostic factors, such as the tumour site or nodal involvement. Ascertaining whether biological factors really provide novel prognostic information and that they are not simply a reflection of the weight of clinical factors is an arduous task.

This case–control study compared the biological characteristics of patients who developed distant metastases with those of patients with a similar tumour site, node size and level, who never developed metastasis. The statistical methodology was feasible as we had at our disposal a large series of 914 patients who had undergone neck surgery in our institution (Mamelle et al, 1994). Patients with metastasis were matched with their metastasis-free counterparts who had the same clinical prognostic factors and the same treatment. A previous study conducted by us had shown that the node size and level in the neck were the factors that best predicted metastases. When the statistical methodology was used to analyse the biological factors selected, histological grading, including differentiation and keratinization, and the presence or absence of vascular emboli detected in biopsy specimens of the primary tumour as well as immunohistochemical expression of Rb and bcl2 were not found to be predictive of distant metastasis. Only two factors appeared to be potentially of prognostic import, namely p53 and c-erb-B2 immunohistochemical expression.

The clinical significance of p53 mutations and expression is currently being investigated in HNSCC. Some studies have noted the absence of a significant correlation between p53 accumulation and clinical outcome (Somers et al, 1992; Bourhis et al, 1994; Ahomadegbe et al, 1995). In a recent paper, Shin et al (1996) found that p53 expression was associated with an increased risk of second primaries and locoregional failures but not with distant metastasis. In our study, the risk of distant metastasis was multiplied by 1.7 in patients with p53 hyperexpression, but this result was not statistically

© Cancer Research Campaign 1998

significant. With 63 pairs of patients, this study had an 80% chance of detecting a relative risk of 2.7 and 95% chance of detecting a relative risk of 3.5. Our results confirm that p53 expression is not a strong prognostic factor for distant metastasis in HNSCC.

The main finding in this case–control study was unexpected: c-erb-B2 immunohistochemical expression was significantly associated with a decreased risk of distant metastases in patients with pharyngeal tumours. This result was initially obtained in the group of patients with oropharyngeal tumours and was confirmed in the group of patients with hypopharyngeal tumours. As immunohistochemical assessment of specimens is rapid, c-erb-B2 expression could be used in routine practice as a biological prognostic tool. It should be emphasized that the interpretation of immunohistochemical analyses is dependent on the quality of the technique (quality of specimen, fixative and sensitivity of c-erb-B2 antibodies) and that quantification of c-erb-B2 expression is contingent on the experience of the pathologist. A polyclonal antibody (DA 485) was used in this study because it had already been tried and tested in our institution in breast cancer (Terrier et al, 1996) and because its sensitivity and specificity had been favourably evaluated in other studies (Press et al, 1994). Membrane staining was only considered because the c-erb-B2 gene product is normally localized in the cell membrane. Cytoplasmic staining in the absence of membrane staining was rare and considered to be non-specific (Craven et al, 1992).

c-erb-B2 amplification and overexpression has been correlated with a shorter surivival in breast cancer (Slamon et al, 1987; Press et al, 1994), yet some reports state that c-erb-B2 is of limited prognostic value, if any (Van de Vijvers, 1988; Zhou, 1989). The prognostic value of c-erb-B2 has not been extensively studied in HNSCC patients. The studies that included patients with tumours of different sites and stages found no correlation between c-erb-B2 expression and survival (Craven et al, 1992; Field et al, 1992). Inconsistent with our results, a recent paper reported a correlation between c-erb-B2 overexpression and poor survival in 39 patients presenting SCC of the oral cavity (Xia et al, 1997). There are, however, many differences between their paper and ours. First, the prognostic value of c-erb-B2 in Xia's paper can be attributed to its relation with clinical factors (nodal involvement, distant metastases at initial presentation). In our work, we compared the occurrence of distant metastases in patients with similar clinical features. Second, Xia et al studied oral SCC and our study only included pharyngeal tumours. C-erb-B2 overexpression may, as suggested by Xia et al, be a characteristic of oral SCC and not of other HNSCC.

c-erb-B2 oncogene abnormalities, including gene amplification and overexpression are a critical event in carcinogenesis in breast tissue. In contrast, c-erb-B2 alterations have never been found at the DNA level in HNSCC, nor has gene activation been proven during HNSCC carcinogenesis (Riviere et al, 1991). Kilpi et al (1995) have compared c-erb-B2 immunohistochemical expression in normal oral mucosa, lichen planus and subsequent squamous cell carcinoma: c-erb-B2 was more frequently expressed in normal mucosa than in tumours. In a small series of patients with pharyngeal carcinoma, we also compared the immunohistochemical expression of c-erb-B2 in tumour with that of non-transformed mucosa surrounding the tumour and obtained similar results. These data suggest that the loss of c-erb-B2 expression, at least in a subset of HNSCC, is a step in the process of tumorigenesis. The combined activity of different oncogenes and loss of activity of tumour-suppressor genes are a prerequisite for the carcinogenic process. A quantitative or qualitative modification in c-erb-B2 expression could be accompanied by activation of other oncogenes. c-erb-B2 and the epidermal growth factor receptor (EGFR) are, to a high degree, homologous and these proteins interact in concert to increase mitogenic signal transduction (Dougall et al, 1993). In human skin (Maguire et al, 1989) and in human renal cell carcinoma (Weidner et al, 1990), c-erb-B2 and EGFR have been shown to exhibit an inverse relationship. In human breast cancer, using a sensitive radioimmunohistochemical assay, Robertson et al (1996) have shown an inverse relationship between EGFR and c-erb-B2 expression, which is disrupted by c-erb-B2 amplification. For a better understanding of the biology of HNSCC, further investigations are warranted on combined alterations of these proteins and/or other oncogenes.

### ACKNOWLEDGEMENTS

This work was supported by CRC 9523 provided by Institut Gustave Roussy. We thank Ms Lorna St Ange for editing the text and Miss Linda Charpentier for preparing the manuscript.

### REFERENCES

Ahomadegbe JC, Barrois M, Fogel S, Le Bihan ML, Douc-Rasy S, Duvillard P, Armand JP and Riou G (1995) High incidence of p53 alterations (mutation, deletion, overexpression) in head and neck primary tumors and metastases: absence of correlation with clinical outcome. Frequent protein overexpression in normal epithelium and in early non-invasive lesions. *Oncogene* **10**: 1217–1227

Bourhis J, Bosq J, Wilson GD, Bressac B, Talbot M, Leridant AM, Dendale R, Janin N, Armand JP, Luboinski B, Malaise EP, Wibault P and Eschwege F (1994) Correlation between p53 gene expression and tumor-cell proliferation in oropharyngeal cancer. *Int J Cancer* **57**: 458–462

Broders AC (1926) Carcinoma: grading and practical application. *Arch Pathol* **2**: 376–381

Craven J, Pavelic Z, Stambrook P, Pavelic L, Gapany M, Kelley D, Gapany S and Gluckman JL (1992) Expression of c-erb-B2 gene in human head and neck carcinoma. *Anticancer Res* **12**: 2273–2276

Dougall WC, Qian X and Greene MI (1993) Interaction of the Neu/p185 and EGF receptor tyrosine kinases: implications for cellular transformation and tumor therapy. *J Cell Biochem* **53**: 61–73

Field JK, Spandidos DA, Yiagnisis M, Gosney JR, Papadimitriou K and Stell PM (1992) C-erb-B2 expression in squamous cell carcinoma of the head and neck. *Anticancer Res* **12**: 613–620

Hsu SM, Raine L and Fanger H (1981) Use of Avidin-Biotin-Peroxidase complex (ABC) in immunoperoxidase techniques: a comparison between ABC and unlabelled antibody (PAP) procedures. *J Histochem Cytochem* **29**: 577–580

Janot F, Klijanienko J, Russo A, Mamet JP, De Braud F, El-Naggar AK, Pignon JP, Luboinski B and Cvitkovic E (1996) Prognostic value of clinocopathological parameters in head and neck squamous cell carcinoma: a prospective analysis. *Br J Cancer* **73**: 531–538

Kilpi A, Rich AM, Konttinen YT and Reade PC (1995) The expression of c-erb-B2 protein in the keratinocytes of oral mucosal lichen planus. *Br J Dermatol* **133**: 847–852

Logothetis C, Xu H, Ro J, Hue S, Sahin A, Ordonez N and Benedict W (1992) Altered expression of retinoblastoma protein and known prognostic variables in locally advanced bladder cancer. *J Natl Cancer Inst* **84**: 1256–1261

Maguire HC, Jaworsky C, Cohen JA, Hellman M, Weiner DB and Greene MI (1989) Distribution of neu (c-erb-B2) protein in human skin. *J Invest Dermatol* **89**: 786–790

Mamelle G, Pampurik J, Luboinski B, Lancar R, Lusinchi A and Bosq J (1994) Lymph nodes prognosis factors in head and neck squamous cell carcinoma. *Am J Surg* **168**: 494–498

Mehta CR and Patel NR (1995) Exact logistic regression: theory and examples. *Stat Med* **14**: 2143–2160

Mizutani T, Onda M, Tokunaga A, Yamanaka N and Sugisaki Y (1993) Relationship of c-erb-B2 protein expression and gene amplification to invasion and metastasis in human gastric cancer. *Cancer* **72**: 2083–2088

Pezzella F, Turley H, Kuzu I, Tungekar MF, Dunnill MS, Pierce CB, Harris A, Gatter KC and Masson DY (1993) Bcl-2 protein in non-small-cell lung carcinoma. *N Engl J Med* **329**: 690–694

Press MF, Hung G, Godolphin W and Slamon DJ (1994) Sensitivity of HER-2/neu antibodies in archival tissue samples: potential source of error in immunohistochemical studies of oncogene expression. *Cancer Res* **54**: 2771–2777

Riviere A, Becker J and Löning T (1991) Comparative investigation of c-erbB2/neu expression in head and neck tumors and mammary cancer. *Cancer* **67**: 2142–2149

Robertson KW, Reeves JR, Smith G, Keith WN, Ozanne BW, Cooke G and Stanton PD (1996) Quantitative estimation of epidermal growth factor receptor and c-erb-B2 in human breast cancer. *Cancer Res* **56**: 3823–3830

Roland JJ, Caslin AW, Nash J and Stell PM (1992) Value of grading squamous cell cardinoma of the head and neck. *Head & Neck* **14**: 224–229

Shin DM, Lee SL, Lippman SM, Lee JJ, Tu NZ, Choi G, Heyne K, Shin HJC, Ro JY, Goepfert H, Hong WK and Hittelman WN (1996) P53 expression: predicting recurrence and second primary tumors in head and neck squamous cell carcinoma. *J Natl Cancer Inst* **88**: 519–529

Slamon DJ, Clark GM, Wong SG, Levin WJ, Ullrich A and McGuire WL (1987) Human breast cancer: correlation of relapse and survival with amplification of the HER-2/neu oncogene. *Science* **235**: 177–182

Somers K, Merrick A, Lopez M, Incognito L, Schechter G and Casey G (1992) Frequent p53 mutations in head and neck Cancer. *Cancer Res* **52**: 5997–6000

Terrier P, Mouriesse H, Loridon B, Gotteland M, May-Levin F and Delarue JC (1996) Use of polyclonal antibody for the determination of the prognostic value of c-erb-B2 protein over-expression in human breast cancer. *Acta Oncologica* **35**: 23–30

Van de Vijver MJ, Perterse JL, Wolter JM, Mooi WJ, Wisman P, Lomans J, Dalesio O and Nusse R (1988) Neu-protein overexpression in breast cancer. Association with comedotype ductal carcinoma in situ and limited prognostic value in stage II breast cancer. *N Engl J Med* **319**: 1239–1245

Weidner U, Peter S, Strohmeyer T, Hussnätter R, Ackermann R and Sies H (1990) Inverse relationship of epidermal growth factor receptor and HER2/neu gene expression in human renal cell carcinoma. *Cancer Res* **50**: 4504–4509

Xia W, Lau YK, Zhang HZ, Liu AR, Li L, Kiyokawa N, Clayman GL, Katz RL and Hung MC (1997) Strong correlation between c-erb-B2 overexpression and overall survival of patients with oral squamous cell carcinoma. *Clin Cancer Res* **3**: 3–9

Yoo G, Xu H, Brennan J, Westra W, Hruban R, Koch W, Benedict W and Sidransky D (1994) Infrequent inactivation of the retinoblastoma gene despite frequent loss of chromosome 13q in head and neck squamous cell carcinoma. *Cancer Res* **54**: 4603–4606

Zhou DJ, Ahuji H and Cline MJ (1989) Proto-oncogene abnormalities in human breast cancer. c-erb-B2 amplification does not correlate with recurrence of disease. *Oncogène* **4**: 105–108

*© Cancer Research Campaign 1998*